# Online Mindfulness-Based Cognitive Behavioral Therapy Intervention for Youth With Major Depressive Disorders: Randomized Controlled Trial

**DOI:** 10.2196/24380

**Published:** 2021-03-10

**Authors:** Paul Ritvo, Yuliya Knyahnytska, Meysam Pirbaglou, Wei Wang, George Tomlinson, Haoyu Zhao, Renee Linklater, Shari Bai, Megan Kirk, Joel Katz, Lillian Harber, Zafiris Daskalakis

**Affiliations:** 1 School of Kinesiology and Health Sciences York University Toronto, ON Canada; 2 Department of Psychology York University Toronto, ON Canada; 3 Department of Psychiatry University of Toronto Toronto, ON Canada; 4 Centre for Addiction and Mental Health Toronto, ON Canada; 5 Temerty Centre for Therapeutic Brain Intervention Centre for Addiction and Mental Health Toronto, ON Canada; 6 THETA Collaborative University Health Network University of Toronto Toronto, ON Canada; 7 Biostatistics Unit University Health Network University of Toronto Toronto, ON Canada; 8 Aboriginal Engagement and Outreach Centre for Addiction and Mental Health Toronto, ON Canada; 9 Department of Psychology University of Toronto Toronto, ON Canada; 10 Department of Psychiatry University of California San Diego La Jolla, CA United States; 11 Mood and Anxiety Ambulatory Services Centre for Addiction and Mental Health Toronto, ON Canada

**Keywords:** intervention study, telemedicine, electronic CBT, clinical trial, depression, cognitive behavioral therapy, CBT, online therapy, online intervention, youth, young adult

## Abstract

**Background:**

Approximately 70% of mental health disorders appear prior to 25 years of age and can become chronic when ineffectively treated. Individuals between 18 and 25 years old are significantly more likely to experience mental health disorders, substance dependencies, and suicidality. Treatment progress, capitalizing on the tendencies of youth to communicate online, can strategically address depressive disorders.

**Objective:**

We performed a randomized controlled trial (RCT) that compared online mindfulness-based cognitive behavioral therapy (CBT-M) combined with standard psychiatric care to standard psychiatric care alone in youth (18-30 years old) diagnosed with major depressive disorder.

**Methods:**

Forty-five participants were randomly assigned to CBT-M and standard care (n=22) or to standard psychiatric care alone (n=23). All participants were provided standard psychiatric care (ie, 1 session per month), while participants in the experimental group received an additional intervention consisting of the CBT-M online software program. Interaction with online workbooks was combined with navigation coaching delivered by phone and secure text messaging.

**Results:**

In a two-level linear mixed-effects model intention-to-treat analysis, significant between-group differences were found for the Beck Depression Inventory-II score (difference –8.54, *P*=.01), Quick Inventory of Depressive Symptoms score (difference –4.94, *P*=.001), Beck Anxiety Inventory score (difference –11.29, *P*<.001), and Brief Pain Inventory score (difference –1.99, *P*=.03), while marginal differences were found for the Five Facet Mindfulness Questionnaire–Nonjudging subscale (difference –2.68, *P*=.05).

**Conclusions:**

These results confirm that youth depression can be effectively treated with online CBT-M that can be delivered with less geographic restriction.

**Trial Registration:**

Clinical Trials.gov NCT03406052; https://www.clinicaltrials.gov/ct2/show/NCT03406052

## Introduction

Approximately 70% of all mental health problems appear before 25 years of age and often become chronic when not treated or ineffectively treated [1]. Such data raise questions about elevated depression rates in youth [2] as exemplified in the National Survey on Drug Use and Health (N=611,880), which found an increase in the depressive episode rates by 63% from 2009 to 2017 [2]. Youthful online engagement makes online intervention delivery attractive, particularly with possible reductions in costs, geographic barriers, and access inequities [3-6].

Cognitive behavioral therapy (CBT) is the best-validated psychotherapy [7], and in recent years has been coordinated with mindfulness meditation (CBT-M) following strong evidence of the combined efficacy [8,9]. Online CBT-M research with student and adult populations has yielded psychometric and neurophysiological [10-20] benefits in single-arm and randomized controlled trials (RCTs). These results join a growing world literature supporting online CBT efficacy, exemplified in a meta-analysis of 3876 RCT participants indicating that online CBT was significantly more effective than control conditions in reducing depressive symptoms (Hedges *g*=0.27) [21]. Individual RCTs have shown online CBT to be equally effective to in-person CBT in studies with large effect sizes, along with substantial remission rates for major depressive disorder [21].

These findings motivated a focus on assessing online CBT in patients concurrently receiving standard psychiatric care to examine whether online CBT *and* psychiatric treatment as usual (TAU) was superior to psychiatric TAU. Control participants received pharmacotherapy only when deemed appropriate by treating psychiatrists, and the TAU comparison accounted for the standard use of and response to antidepressant medications.

The key behavioral intervention in this study was online access to 24 CBT workbooks and 56 mindfulness instruction videos that supported metacognitive change and autonomic balance [22], which have been linked to improved mood and reduced anxiety [23]. Navigation coaching was supplied by students who were pursuing graduate degrees (MSc, MA, PhD) in kinesiology and health science, education, and psychology. Their group training (prior to and during the study) took place at a seminar (for 1.5 hours weekly) that focused on reviews of CBT and mindfulness-based clinical research supplemented by anonymized case discussions. One (cumulative) hour of coaching was provided to each participant weekly during 24 weeks (which included text-message exchanges with participants), and each coach received 1-hour weekly sessions of one-to-one supervision.

Navigation coaching has been increasingly applied to support the adoption of evidence-based, health-related behaviors as demonstrated in adherence to cancer screening, and exercise and diet regimens [24]. Our experiences with navigation coaching include assisting patients with type 2 diabetes to reduce hemoglobin A1c blood levels [18-20] and assisting individuals in undertaking colorectal cancer screening [25]. Positive outcomes suggest that navigation coaching can be applied to the treatment of depression in assisting the use of CBT and mindfulness methods to address depressive symptoms [26]. Past successes with text messaging–assisted interventions (with critical medical outcomes) [18-20] further influenced the emphasis on text messaging between navigator coaches and patients.

Our study objective was to assess whether online CBT-M with weekly interactions with a coach navigator and standard psychiatric care was superior to standard psychiatric care alone (as workbooks and videos were never provided without coach navigator assistance). We hypothesized that intervention participants would demonstrate significant improvements in primary outcomes when compared to the waitlist controls who received only standard psychiatric TAU.

## Methods

### Design and Recruitment

The study was approved by the Research and Ethics Boards of the Centre for Addiction and Mental Health (CAMH; Protocol Reference number 115/2016-01) and York University (Certificate number 2017–154) in Toronto, Canada, and was registered at ClinicalTrial.gov (NCT03406052). This included distinct software platform approval for all privacy and security requirements at CAMH. The study evaluated the efficacy of CBT-M to treat young adults (18-30 years of age) with major depressive disorder. Participants were identified from service wait lists at the CAMH by research coordinators and in the prescreening of new clinic referrals. The investigative team was informed about possible participant eligibility and the client’s clinician was notified. The clinician then asked the client if she/he was willing to meet with a study team member to explore participation. Information about the study was only shared once the clients agreed to meet for potential participation. A biostatistician (GT) performed electronic randomization of participants, assigning study IDs to intervention vs waitlist control participants. Information regarding each study ID with its respective group allocation was transferred onto cards placed in individually sealed, opaque envelopes. After a participant completed baseline questionnaires, the research coordinator opened the next envelope in the sequence to assign the group and respective study ID.

**Figure 1:**
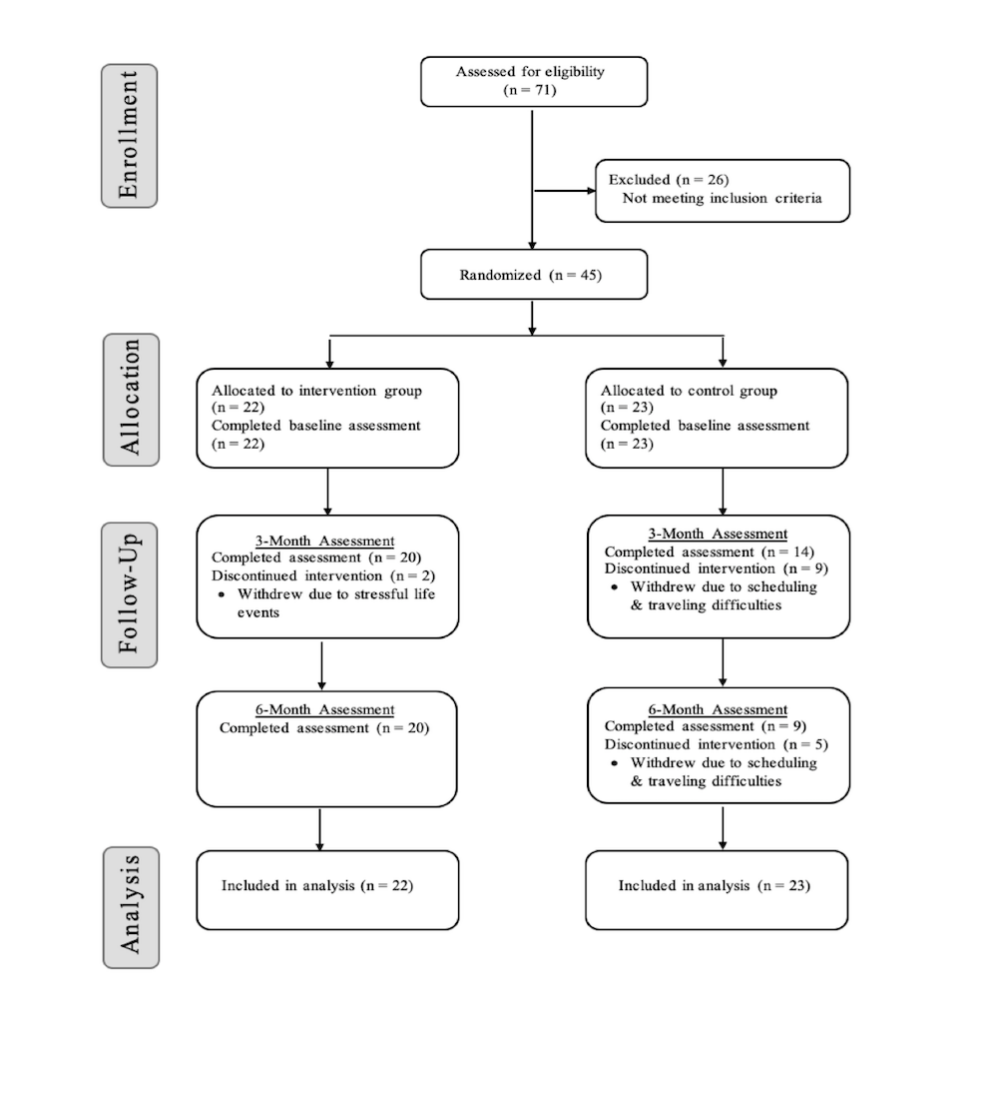
Consort flow diagram.

The inclusion criteria were: (1) aged 18 to 30 years; (2) Beck Depression Inventory-2 (BDI-II) score of at least mild severity, with no upper limit (BDI-II score≥14) [27]; (3) Mini-International Neuropsychiatric Interview (MINI)-confirmed psychiatric diagnosis of major depressive disorder [28]; and (4) fluent in English. All patients were diagnosed by a CAMH physician, with diagnoses confirmed using the MINI interview administered at the screening visit. The exclusion criteria were: (1) individuals who were currently receiving weekly structured psychotherapy; (2) individuals who met the Diagnostic and Statistical Manual of Mental Disorders-V criteria for severe alcohol/substance use disorders in the past 3 months, individuals who demonstrated clinically significant suicidal ideation (defined as imminent intent), and individuals who had attempted suicide in the past 6 months; and (3) individuals with comorbid diagnoses of borderline personality, bipolar disorder, schizophrenia, and/or obsessive compulsive disorder (Figure 1).

### Intervention

All participants were provided standard psychiatric care, operationally defined as one monthly session of TAU and mostly pharmacotherapy focused. Experimental participants received the additional CBT-M program content (workbooks and videos) accessed online through a software platform developed by NexJ Health, Inc. The platform, NexJ Connected Wellness (NCW), has unique properties that facilitated participant use. Interactions with the online workbooks were combined with navigation coaching (total 24-hour duration over 6 months), delivered as phone and text message exchanges. Each participant was also given a Fitbit-HR Charge 2 that assessed physical steps and 24-hour heart rate in 5-second durations combined with the NexJ Health, Inc software that permitted daily monitoring.

The intervention content was built on two prior successful web-based CBT-M RCTs with students [10-17] and on effective methods used with other populations assessed in RCTs [29-39]. The online content included 24 workbook chapters reflecting multiple topics (eg, Living by Your Truths, Overcoming Wired-ness and Tired-ness, Mindfulness and Relationships, Loss and Grief, Resilience, Befriending Ourselves, Befriending Your Body With Exercise, Body Image and Mindfulness, Intimacy, Forgiveness, Overcoming Procrastination, Dealing With Negative Moods, Stress Resilience, Overcoming Performance Anxiety, and Cultivating Inspiration). The content was covered sequentially on a weekly basis with navigator coach guidance. In summary, key intervention features were 24-hour access and CBT-M contents that addressed specific symptoms and generic depressive experiences. The online platform used is produced and maintained by NexJ Health, Inc in Toronto, Ontario, and is the same basic platform employed in a prior study [19], although it has been upgraded numerous times in the interim. NexJ Health, Inc provided use of the NCW platform free of charge (as a research partner) but contributed no other funding or support for the study.

### Outcome Measures

The primary outcome measure was the BDI-II [27], and the secondary outcomes focused on anxiety (Beck Anxiety Inventory [BAI]) [40], depression (Quick Inventory of Depressive Symptomatology [QIDS]) [41], the 24-item Hamilton Depression Rating Scale (HDRS-24; assessed by a blinded interview rater) [42], mindfulness (5-Facet Mindfulness Questionnaire [FFMQ]) [43], and pain (Brief Pain Inventory [BPI]) [44].

All self-report measures and the HDRS-24 interviews were conducted at the same CAMH Ambulatory Service setting. The HDRS-24 interview rater was blinded to intervention and control conditions for the trial duration.

### Statistical Analysis

We used a two-level linear mixed-effects model to compare the difference in the rate of change regarding outcome scores between the intervention and control groups, accounting for the repeated measurement nature of the data. A full information maximum-likelihood method was used to deal with missing data [45]. Age, sex, and ethnicity were further included as auxiliary variables for this approach.

## Results

### Analyses

Data obtained from participants during study visits were deidentified and stored as electronic case reporting forms (CRFs) on the CAMH REDCap system, with the CRF paper copies stored in a secure, locked cabinet. Participant characteristics are summarized via descriptive statistics in Table 1. Group equivalence at baseline in terms of demographic and clinical variables was confirmed.

**Table 1:** Baseline demographic characteristics of study participants.

Characteristics			CBT-M^a^ (n=22)	WLC^b^ (n=23)	P value
Age (years), mean (SD)			25 (3.319)	24 (3.233)	.41
**Gender, n (%)**					.30
		Male	10 (46)	7 (30)	
		Female	12 (54)	16 (70)	
**Ethnicity, n (%)**					.57
		Caucasian	13 (59)	12 (52)	
		Asian	6 (27)	4 (17)	
		African-American	0 (0)	1 (4)	
		Indigenous	0 (0)	1 (4)	
		Other	3 (14)	5 (22)	
**Relationship status, n (%)**					.51
		Married	1 (5)	0 (0)	
		Single	20 (90)	21 (91)	
		Other (eg, common law)	1 (5)	2 (9)	
**Offspring, n**			0 (0)	0 (0)	N/A^c^
**Work status, n (%)**					.47
		Employed	12 (55)	15 (65)	
		Not employed	10 (45)	8 (35)	
**Depression duration, mean (SD)**					
	Depression since onset age (years)		17 (4.13)	17 (5.01)	.98
	Duration of current/last depressive episode (months)		9 (14.60)	19 (30.86)	.19
	Number of identified depressive episodes		5.5 (5.06)	6.1 (6.94)	.72
**Psychiatric history, mean**					
	Previous medication trials and failures		1.09	1.21	.91
	Level of substance dependency or abuse		0	0	N/A
	Number of suicide attempts (from MINI^d^), mean		0	0	N/A
**Comorbidities, mean**					
	Psychiatric comorbidities		3.09	3.26	.77
	Physical comorbidities		1.59	1.70	.81
**Outcomes, mean (SD)**					
	Baseline BDI-II^e^		30 (8.40)	27 (7.90)	.21
	Baseline BAI^f^ (mean)		29 (8.53)	22 (9.40)	.008
	Baseline BPI^g^ (average pain x/10)		1.9 (2.50)	1.6 (2.25)	.64
	HDRS^h^		26 (6.96)	26 (6.43)	.96
	QIDS^i^		16 (4.30)	15 (3.70)	.44
	FFMQ^j^-Observing		15 (3.30)	13 (3.54)	.21
	FFMQ**-**Describe		15 (5.25)	14 (3.94)	.45
	FFMQ-Act Aware		12 (3.81)	14 (3.24)	.08
	FFMQ**-**Nonjudging		11 (4.03)	13 (3.52)	.09
	FFMQ**-**Nonreactivity		12 (3.25)	11 (3.44)	.32

^a^CBT-M: mindfulness-based cognitive behavioral therapy.

^b^WLC: waitlist control.

^c^N/A: not applicable.

^d^MINI: Mini-International Neuropsychiatric Interview.

^e^BDI-II: Beck Depression Inventory-2.

^f^BAI: Beck Anxiety Inventory.

^g^BPI: Brief Pain Inventory.

^h^HDRS: Hamilton Depression Rating Scale.

^i^QIDS: Quick Inventory of Depressive Symptomatology.

^j^FFMQ: 5-Facet Mindfulness Questionnaire.

### Primary and Secondary Outcomes

A mean of 4.7 participants were enrolled per month. The intervention and TAU retention differed markedly as 91% (20/22) of intervention participants were retained compared to only 39% (9/23) of the TAU group (at the end of the trial). Of the 14 control dropouts, 9 dropped out shortly after the baseline assessment and 5 dropped out following completion of midterm, 3-month measures. Of the 2 intervention dropouts, both dropped out shortly after enrollment (prior to 3-month measures). The between-group retention differences were significant at 3 months (P=.04) and 6 months (P=.001).

In the two-level linear model intention-to-treat analysis, between-group BDI-II, QIDS, BAI, BPI, and FFMQ–Nonjudging subscale differences were statistically significant (Table 2).

In the within-group differences, participants who completed the intervention (n=20) demonstrated significant reductions in depressive and anxiety symptoms as measured by changes in BDI-II (P<.001), BAI (P<.001), QIDS (P<.001), and (blinded) HDRS (P<.001) scores from pre- to postintervention (Table 3). The effect sizes were very large for the BDI-II (Cohen *d*=1.90 and Hedges *g*=1.82) and large on the QIDS (Cohen *d*=1.43 and Hedges *g*=1.38). All effect sizes were large and two were at or above 1.6 (Cohen *d*), typically calculated as two times a large effect size.

**Table 2:** Between-group differences based on intention-to-treat analysis (N=45).

Outcome	Pretreatment, mean (SD)		Final assessment, mean (SD)		Difference^a^	P value
	Intervention	Control	Intervention	Control		
BDI-II^b,c^	30.14 (8.397)	27.00 (7.909)	13.6 (9.73)	19.78 (16.642)	–8.54	.01
BAI^d,e^	29.14 (8.532)	21.74 (9.401)	15.45 (9.145)	21.67 (15.248)	–11.29	.001
QIDS^e,f^	15.50 (4.307)	14.57 (3.703)	8.95 (4.925)	12.89 (6.03)	–4.94	.001
HDRS^e,g^	26.27 (6.964)	26.17 (6.436)	14.75 (8.735)	22.67 (13.134)	–5.52	.09
BPI^e,h^	1.89 (2.465)	1.553 (2.258)	1.11 (1.753)	2.17 (2.246)	–1.99	.03
FFMQ^i^–Nonjudging^e^	11.23 (4.035)	13.17 (3.525)	14.95 (5.042)	13.56 (5.199)	2.68	.049
FFMQ–Describing^e^	15.27 (5.248)	14.22 (3.942)	18.45 (4.032)	16.11 (4.314)	0.62	.61
FFMQ–Observing^e^	14.55 (3.306)	13.26 (3.454)	15.15 (3.829)	14.22 (4.738)	–1.22	.16
FFMQ–Awareness^e^	12.33 (3.816)	14.13 (3.238)	16.2 (4.396)	15.56 (4.667)	2.16	.11

^a^Difference of rate of change: a negative value indicates greater reduction in the intervention group.

^b^BDI-II: Beck Depression Inventory-2.

^c^Planned analysis of primary outcome.

^d^BAI: Beck Anxiety Inventory.

^e^Bonferroni correction was not applied for secondary outcomes.

^f^QIDS: Quick Inventory of Depressive Symptomatology.

^g^HDRS: Hamilton Depression Rating Scale.

^h^BPI: Brief Pain Inventory.

^i^FFMQ: 5-Facet Mindfulness Questionnaire.

**Table 3:** Within-group differences of intervention participants who completed the trial (N=20).

Scale		Change (95% CI)	P value	Cohen *d*
BDI-II^a^		–15.6 (–2.02 to –11.1)	<.001	1.9
BAI^b^		–127 (–16.9 to –8.5)	<.001	1.5
QIDS^c^		–6.2 (–8.3 to –4.0)	<.001	1.4
HDRS^d^		–10.7 (–14.7 to –6.6)	<.001	1.6
BPI^e^		–0.8 (–1.8 to 0.3)	.14	—^f^
**FFMQ** ^g^				
	Observing	0.01 (–0.7 to 0.9)	.46	—
	Describing	2.5 (0.5-4.5)	.02	1.5
	Awareness	2.8 (1.3-4.3)	.001	1.4
	Nonjudging	2.5 (0.9-4.2)	.005	1.4

^a^BDI-II: Beck Depression Inventory-2.

^b^BAI: Beck Anxiety Inventory.

^c^QIDS: Quick Inventory of Depressive Symptomatology.

^d^HDRS: Hamilton Depression Rating Scale.

^e^BPI: Brief Pain Inventory.

^f^—:not applicable.

^g^FFMQ: 5-Facet Mindfulness Questionnaire.

## Discussion

The online CBT-M intervention was beneficial, given significant between-group differences in depression (BDI-II, QIDS), anxiety (BAI), pain (BPI), and mindfulness (FFMQ–Nonjudging subscale). Other notable between-group observations involved a 9% dropout rate in the intervention group that significantly differed from the 61% dropout rate in the TAU control group (the 61% dropout rate was estimated at ~14% above the mean for CAMH TAU). This difference suggests that the intervention had positive effects on participant retention. The intervention sample included a large subgroup with severe depression (n=10 participants, defined as severe by a BD-II score>29), 50% of whom were in remission (BDI<14) at the final (6-month) assessment. Of the 6 participants who exhibited moderate depression, 5 achieved remission, and of the 3 study participants with mild depression, 2 achieved remission.

Given the CBT-M intervention, it was notable that between-group differences were found in the Nonjudging subscale of the FFMQ that assesses the excess self-critical thinking associated with distress [43]. Intervention group participants engaged in significantly less self-critical self-judgment at the 6-month follow up than the TAU controls. Although the study sample size did not allow for mediation analyses [46], the between-group difference observed suggests that the mindfulness component of the CBT-M intervention was likely involved in the modification of depressogenic cognitions [2]. The between-group differences also appear linked to the self-acceptance emphasis in the CBT-M interventions employed.

Significant between-group differences were found in self-reported chronic pain as indicated in the BPI scale. The inclusion of a pain assessment reflects recent findings about the high comorbidity prevalence in depression with respect to chronic pain [47] and the possible efficacy of behavioral pain reduction methods [48,49]. Despite this study’s findings, the behavioral intervention literature on reductions in chronic pain remains sparse, and additional targeted studies in populations with pain and mental health difficulties are warranted.

Although attrition in psychiatric treatment has been linked to early improvements associated with medication changes [50], this explanation does not seem to apply in the current trial, as 4 of the 5 total control participants who discontinued after the 3-month midterm measures received no pharmacotherapy or no modifications in the prestudy pharmacotherapy established. The control participants who dropped out following the baseline assessment (before midterm, 3-month assessment; n=9) did not receive medication initiation or modification. The attrition difference also did not appear to be based on more severe baseline depression as the mean BDI-II depression score at baseline for TAU control participants (BDI-II=27.0) reflected milder depression symptoms than those of intervention participants (mean BDI-II=30.14).

Significant study strengths included the control comparison with a standard-care psychiatry intervention, delivered at the same institution, independently versus in combination with the experimental behavioral intervention. This resulted in detailed records of how pharmacological and behavioral interventions interacted, assisting estimations of independent and combined benefits. The study further controlled for the intervention-related placebo effects observed in 35%-40% of RCT participants exposed to TAU conditions [7]. This was also a necessary control for medication effects, given that individuals treated for depression show improvement with antidepressants alone [7]. In this study, CBT-M effects were clearly additive to TAU effects. Although the TAU-only group attrition rate can be seen as a study limitation, there were demonstrated associations between the CBT-M intervention and retention (ie, lower attrition in the experimental group) that indicated retention benefits associated with the behavioral treatment.

In recent meta-analyses focused on CBT-M delivery for depression, multiple CBT modalities have been assessed, notably individual, group, telephone-based, and guided self-help, all of which appear to be significantly more effective than waitlist and care-as-usual control conditions, and unguided self-help [51]. These analyses reflect the investigative search for the most cost-effective CBT delivery. In the context of current meta-analyses, our intervention can be characterized as combining telephone-based with guided self-help (online), with results that show significantly better outcomes than care-as-usual controls.

Key limitations of our study include a lack of participant blinding and the limited power associated with a small sample size. However, the HDRS assessment was undertaken by a rater blinded to group allocation. Although the between-group differences on the HDRS were trending toward significance (P=.09), they were not statistically significant. A final limitation is that the study psychiatrists administering TAU to control and intervention participants were not blind to which participants were in the intervention versus control groups, and this might have led to biased treatment.

Future studies comparing CBT-M and standard-care psychiatry would benefit from larger sample sizes, more complete blinding, and extended follow up after intervention conclusion (eg, 6-12 months). Despite these limitations, the results indicate that online CBT-M combined with TAU psychiatric treatment was an effective treatment for major depressive disorder and led to significantly greater reductions in BDI-II scores than TAU psychiatry alone.

## Appendix

Multimedia Appendix 1 CONSORT-eHEALTH checklist (V 1.6.1).

## Abbreviations

BAI: Beck Anxiety Inventory
BDI: Beck Depression Inventory
BPI: Brief Pain Inventory
CAMH: Centre for Addiction and Mental Health
CBT: cognitive behavioral therapy
CBT-M: mindfulness-based cognitive behavioral therapy
CIHR: Canadian Institutes of Health Research
CRF: case reporting form
FFMQ: 5-Facet Mindfulness Questionnaire
HDRS-24: 24-item Hamilton Depression Rating Scale
MINI: Mini-International Neuropsychiatric Interview
NCW: NexJ Connected Wellness
QIDS: Quick Inventory of Depressive Symptomatology
RCT: randomized controlled trial
TAU: treatment as usual
